# Glutathione-Garlic Sulfur Conjugates: Slow Hydrogen Sulfide Releasing Agents for Therapeutic Applications

**DOI:** 10.3390/molecules20011731

**Published:** 2015-01-20

**Authors:** Ashif Iqbal Bhuiyan, Vilma Toska Papajani, Maurizio Paci, Sonia Melino

**Affiliations:** 1 Department of Chemical Sciences and Technologies, University of Rome Tor Vergata, via della Ricerca Scientifica, Rome 00133, Italy; E-Mails: ashif.biochim@gmail.com (A.I.B.); paci@uniroma2.it (M.P.); 2 Department of Professional Chemistries and Pharmacognosy, University of Medicine, Tirana, Str. “Dibra”, Nr. 371, Tirana 1000, Albania; E-Mail: toskavilma@gmail.com

**Keywords:** garlic, hydrogen sulfide, allyl sulfur compounds, sulfurtransferase, tumor cells, extraction, sulfur conjugates

## Abstract

Natural organosulfur compounds (OSCs) from *Allium sativum* L. display antioxidant and chemo-sensitization properties, including the* in vitro* inhibition of tumor cell proliferation through the induction of apoptosis. Garlic water- and oil-soluble allyl sulfur compounds show distinct properties and the capability to inhibit the proliferation of tumor cells. In the present study, we optimized a new protocol for the extraction of water-soluble compounds from garlic at low temperatures and the production of glutathionyl-OSC conjugates during the extraction. Spontaneously, Cys/GSH-mixed-disulfide conjugates are produced by* in vivo* metabolism of OSCs and represent active molecules able to affect cellular metabolism. Water-soluble extracts, with (GSGaWS) or without (GaWS) glutathione conjugates, were here produced and tested for their ability to release hydrogen sulfide (H_2_S), also in the presence of reductants and of thiosulfate:cyanide sulfurtransferase (TST) enzyme. Thus, the TST catalysis of the H_2_S-release from garlic OSCs and their conjugates has been investigated by molecular *in vitro* experiments. The antiproliferative properties of these extracts on the human T-cell lymphoma cell line, HuT 78, were observed and related to histone hyperacetylation and downregulation of GAPDH expression. Altogether, the results presented here pave the way for the production of a GSGaWS as new, slowly-releasing hydrogen sulfide extract for potential therapeutic applications.

## 1. Introduction

The health benefits of *Allium* species are widely attributed to enzyme transformed organosulfur compounds (OSCs), named thiosulfinates, which are the dominating organosulfur species in fresh garlic and onion tissue macerates, representing between 50% and 94% of the organo-compounds [1]. These compounds exhibit intriguing biological properties: antimicrobial, antioxidant [2,3] and anti-inflammatory actions [4] that are able to upregulate detoxification/cytoprotective enzymes [5,6], such as quinine reductase [1,7] and GST [8,9]. This results in enhancing the chemo-sensitivity in cancer cells, as well as in inducing apoptosis in cultured human cancer cells and causing cell cycle arrest at the G_2_/M or G_1_/S checkpoints [5]. *Allium* OSCs, either alone or in association with other antitumor drugs, are therefore potential candidates as ideal agents for anticancer therapy; although the underlying antitumorigenic mechanism [10,11,12] still requires further clarification at the detailed biochemical level. Very likely, the rate of metabolic clearance of allyl sulfur groups from individual cells is a determinant of the biological response. Thiosulfinates have a half-life of about 1 min [13] in blood, and the presumed fate is through the reaction with thiols in plasma or blood cells. The biochemical transformations of these compounds within the cell and the resulting adducts with the thiol functional groups of the proteins could constitute a relevant event to uncover their anticancer properties. They can freely permeate cell membranes and rapidly react with Cys or GSH to form intracellular Cys/GSH mixed-disulfide conjugates. Cys/GSH-mixed-disulfide conjugates are among the derivates that can be formed from *Allium sativum* L. tissue constituents and* in vivo* metabolism. Very likely, Cys/GSH conjugates can also accumulate and act in different extra-cellular compartments. The understanding of the biological effects of allyl-disulfide conjugates may be, therefore, the key to elucidating the health benefits of *Allium* species. *Allium* OSCs contain sulfur, which is reactive as sulfane sulfur, that has important functions in cells and, very likely, is the mediator of* in vivo* H_2_S signaling [14,15]. As H_2_S-donors, the garlic OSCs release this gasotransmitter with a relatively slow mechanism, which requires the cooperation of endogenous thiols, such as GSH, as described by Knaus* et al.* [16]. The list of the physiological effects mediated by H_2_S has been rapidly expanded, as recently reviewed [17,18,19]. This endogenous gaseous molecule is coupled to a transduction mechanism that accounts for many important biological effects, and its pharmaco-dynamic feature discloses attractive perspectives for the potential pharmaco-therapeutic usefulness of drugs acting on the H_2_S pathway. The inefficient endogenous biosynthesis of this gaseous cellular messenger can lead to a range of diseases and alterations of the cardiovascular, ocular, skeletal and central nervous systems, and it has been associated with Down syndrome, neuroblastoma, hepatoblastoma and celiac disease [20,21]. The correlation of clinical pathologies, such as homocystinuria, cystathioninuria, mercaptolactate-cysteine disulfiduria diseases, due to a deficiency in cystathionine-β-synthase (CBS), cystathionine-γ-lyase (CSE) and 3-mercapto-piruvate-sulfurtransferase (MST) enzymes, which are involved in the H_2_S-generating routes in mammals, with the levels of this gasotransmitter is noteworthy [17,18,19,20,21]. Moreover, also the human cyanide:thiosulfate sulfurtransferase enzyme (TST, rhodanese EC. 2.8.1.1), similarly to MST-bound persulfide, sharing ~60% sequence similarity with it, is able to catalyze the H_2_S formation [22,23]. Although this mitochondrial enzyme catalyzes H_2_S production and it is also involved in the metabolism of OSCs, at this moment, the role of the TST in the release of the gasotransmitter by OSCs in mitochondria is not clear. Accordingly, here, we decided to investigate the effects of the TST on the H_2_S-release from garlic OSCs and their conjugates* in vitro*.

Several studies have suggested a great therapeutic potential for exogenous sources of H_2_S as powerful innovative pharmaco-therapeutic agents against widely-diffused cardiovascular and neurodegenerative pathologies, including Parkinson’s and Alzheimer’s disease [17,24,25]. However, the administration of gaseous H_2_S is greatly limited by the difficulty of ensuring an accurate posology control and the risk of overdose. Ideally, H_2_S-donors for therapeutic purposes should generate H_2_S with controlled slow releasing rates; this pharmacological feature seems to be exhibited by garlic OSCs. In the present investigation, glutathione-garlic OSC conjugates (GS-OSCs) have been also synthesized using a new extraction protocol at a low temperature. The extracts have been characterized by RP-HPLC, ^1^H-NMR spectroscopy and LC-mass spectrometry and assayed for their ability to produce the gasotransmitter, H_2_S, and to affect the activity and expression of enzymes with catalytic cysteines. This last effect has been investigated both* in vitro*, using as a model a bacterial TST enzyme, which is characterized by the presence of only one cysteine present in the catalytic site, and* in vivo*, using cancer cells, evaluating the expression of the glycolytic enzyme, glyceraldehyde 3-phosphate dehydrogenase (GAPDH). Accordingly, an antiproliferative effect of the garlic water-soluble extracts (GaWS and GSGaWS) on the human T-cell lymphoma cell line, HuT 78, has been observed. In particular, the data reported here also show a reduced pro-apoptotic effect by the presence of GS-OSCs in the garlic extracts, promoting the arrest of the cell cycle in the G_2_/M phase.

## 2. Results and Discussion

### 2.1. Production and Characterization of GS-OSCs from Garlic at a Low Temperature

**Figure 1:**
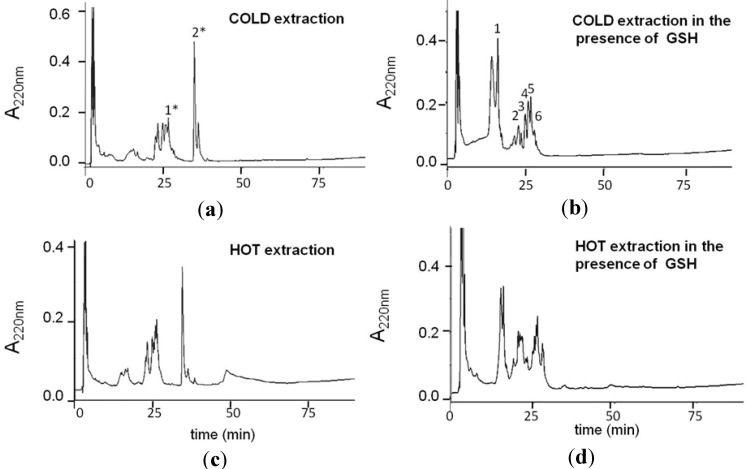
RP-HPLC of the water extracts obtained in the absence (**a**,**c**) or in the presence of GSH (**b**,**d**) obtained using low (a,b) and high (c,d) temperatures. The elution was performed with a linear gradient of 80% CH_3_CN, 0.1% TFA.

Heating during extraction, storage at high temperature and/or extreme pHs can adversely affect the quality of garlic OSCs [26,27,28,29,30,31]. In order to reduce the garlic OSCs’ oxidative and degradation processes that usually occur during the extraction protocols performed at high temperatures, in this study, the preparation of garlic extracts at low temperatures and physiologic pH has been optimized. The water-soluble garlic extracts were, thus, obtained using two different protocols of preparation: one incubating the crushed garlic in a bain-marie at 100 °C and the other one freezing and pressing the crushed garlic at low temperatures obtained using liquid nitrogen. Extracted solutions were then filtered and analyzed by RP-HPLC (Figure 1) and NMR spectroscopy (see Supplementary Material Figure S1). The RP-HPLC analysis of extracts performed at low temperatures showed a better resolution of the elution peaks, very likely due to a minor OSC oxidation in the extraction phase, which is consistent with the presence of a few oxidized forms with limited hydrophobic differences. Moreover, considering the high concentration of GSH in eukaryotic cells, its effects on the formation of bioactive OSCs from allicin was evaluated. The chromatograms of the both water-soluble fractions, which were extracted in the presence of GSH, showed more hydrophilic peaks that were eluted at lower retention times (Figure 1). GS-OSCs were produced during the OSCs extraction, and the analysis of the peaks was performed using ^1^H-NMR spectroscopy and LC-MS spectrometry (see Supplementary Material Figure S1). These analyses allowed us to identify the main OSC conjugates present in the water-soluble glutathione-garlic extract (GSGaWS), as summarized in Table 1. In the chromatograms of the water-soluble garlic extract (GaWS), moreover, we detected a peak, named 2*, in the water-soluble garlic extract (GaWS), corresponding to allicin [C_6_H_10_S_2_O + H]^+^ 163.0 *m/z.* This peak is not present in a detectable concentration in the GSGaWS extract, probably due to the formation of GS-OSCs by the reaction of the allicin with GSH, which leads to the formation of mixed disulfide S-allylmercaptoglutathione (GSSA) [32]. GSSA has been previously described as obtained by the reaction of the water-soluble compound, 2-propenyl thiosulfate (2-PTS), and GSH at physiologic pH [12]. The presence of GSSA (Peak 6), with molecular mass 380.2 *m/z* [C_13_H_21_N_3_O_6_S_2_+H]^+^, in the GSGaWS extract was detected by LC-mass and ^1^H-NMR analyses (see Supplementary Material Figure S1).

**Table 1:** Molecular masses of the glutathionylated-compounds present in the GSGaWS cold extract.

RP-HPLC Peak	Molecular Mass	Compounds in GSGaWS Cold Extract
**1**	614.1 *m/z*	GSSG
**2**	354.1 *m/z*	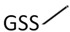
**3**	348.1 *m/z*	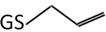
**6**	380.2 *m/z*	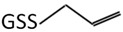

GSSA is a sulfur compound capable of inhibiting the generation of peroxides in fetal brain slices with an antioxidant activity similar to GSH [32] and with antitumor properties [12]. Peak 1* of GaWS and Peaks 4 and 5 of GSGaWS show a molecular mass of 291.1 *m/z* that corresponds to the γ-glutamyl-S-allyl-L-cysteine, a water-soluble compound derived from cysteine that is transformed in S-allyl-L-cysteine (SAC). The latter determines diverse potential clinical benefits, including prevention of the formation of advanced glycation end-products [33], antioxidant activity [34], anticancer [35], anti-hepatotoxic [36] and neurotrophic activity [37].

### 2.2. Effects of the Garlic-WS Obtained at a Low Temperature on the TST Activity

**Figure 2:**
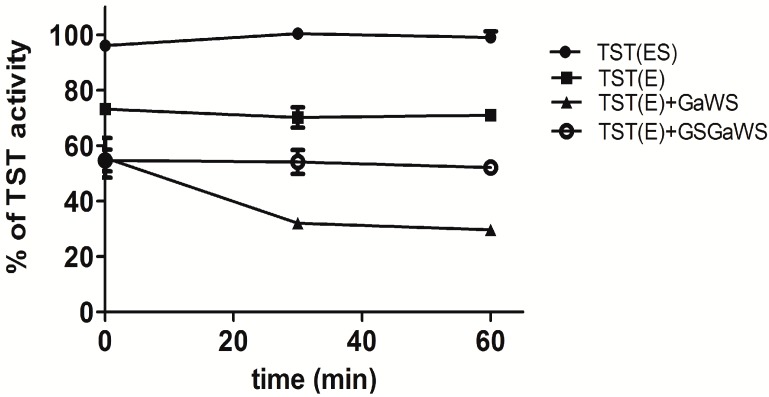
Inhibition of the TST-E form by water-soluble garlic extracts. The solutions were performed by addition at 11.1 μM TST enzyme in 30 mM Tris-HCl buffer, pH 7.5, with or without 6.6 mM KCN, and 10 μL of the GaWS or GSGaWS extracts. Five microliters of the solutions were taken from the solutions after 0, 30 and 60 min for assaying the TST activity.

The regulation of programmed cell death by allyl sulfur compounds, hence on its inhibition of cancer progression, has been related to several epigenetic changes. In keeping with this, a direct inhibitory action of allyl sulfur compounds from garlic on enzymes involved in the detoxification system and in the control of the cell redox state (e.g., GST, sulfurtransferases,* etc.*) is relevant in their complex mechanism of action. The anticancer properties of the allyl sulfur compounds may be related to both their biochemical intracellular transformation and their direct reactivity with thiol groups on redox-sensitive and detoxification proteins. Sulfane sulfur-mediated modification or thioalkenylation of these reactive centers by several garlic allyl sulfur compounds [5,11,14,15,38] may be an important mechanism affecting protein degradation, subsequently inducing cell death. Thus, the effects of the GaWS and GSGaWS extracts obtained at a low temperature on the TST activity were analyzed* in vitro* over time. Although the biological role of the TST enzyme remains still elusive, it represents a link between the cyanide detoxification system and OSC metabolism. The TST enzyme is a ubiquitous enzyme responsible for the biotransformation of cyanide to thiocyanate in every organism [39,40,41,42]. It is involved in a variety of physiological functions starting from the cyanide detoxification to the biogenesis of iron-sulfur clusters [43], transport mechanisms of sulfur/selenium in biologically-available forms [44,45] and sulfide oxidation pathways [46]. The enzyme is characterized by the presence of the rhodanese homology domain (RHOD), a highly-conserved domain present in several classes of proteins (at least 500 proteins) in organisms ranging from archaea to humans, and also notably present in phosphatases of the CDc25 family, which help to regulate the cell cycle [47]. Among others, TST from *A. vinelandii* has only one Cys residue, which is also the catalytic center of the active site of the enzyme; thus, it could represent a good model to study the effects of sulfur compounds on proteins with active thiol groups and with RHOD domain. Figure 2 shows the activity of the recombinant TST enzyme from *A. vinelandii* after incubation of the enzyme with the garlic-WS extracts. These garlic fractions did not affect the TST activity of the persulfurated form (ES), while they were able to inhibit the enzyme activity of the de-persulfurated form of the enzyme (E), obtained by the addition of cyanide (KCN). In particular, major inhibitory effects were obtained after incubation in the presence of the GaWS fraction, indicating that GSGa conjugates may reduce the inhibitory effects on the cyanide detoxification cellular system and the thiolation of cysteine residues of the proteins due to OSCs. These results are in agreement with those previously observed for the 2-PTS and 2-PTS/GSH mixture, which binds the sulfur-free form (E) of TST, inhibiting its TST activity by thiolation of the catalytic cysteine. On the contrary, the purified GSSA was not able to significantly inhibit the TST activity* in vitro* [11,12]. Thus, the present data suggest that the assessable protein cysteine residues may be thioalkenylated by the GaWS extract, while the use of GSGa conjugates or their endogenous formation in cells with a high concentration of GSH reduces this effect, promoting a different and more selective mechanism of action on proteins and the formation of other active sulfur metabolites, such as H_2_S.

### 2.3. H_2_S Production from Garlic Extracts

**Figure 3:**
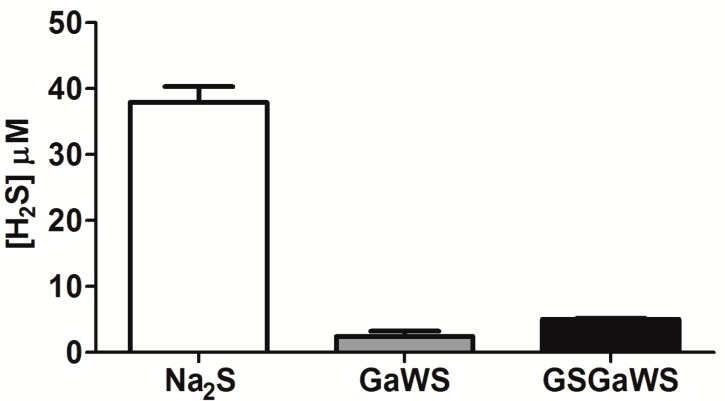
H_2_S production by 25 μL of GaWS (0.8 mg) and GSGaWS (1.1 mg) cold extracts in the absence of reductant after 30 min of incubation at 37 °C, and a 52 μM Na_2_S solution was used as a reference.

**Figure 4:**
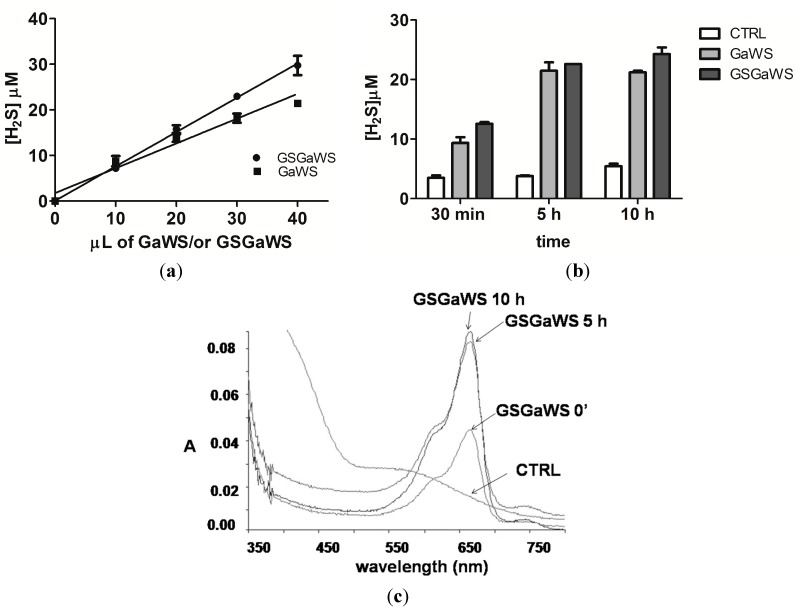
H_2_S production by garlic-WS extracts (cold extractions) obtained (**a**) at different concentrations of GaWS and GSGaWS; (**b**) H_2_S formation over time by 5 μL of GaWS (0.16 mg) and of GSGaWS (0.22 mg) in the presence of 4 mM DTT; (**c**) UV-vis spectra of the solution after 30 min, 5 h and 10 h of incubation at 37 °C. Each bar represents ±SD of three or five experiments.

The endogenous gasotransmitter, H_2_S, can be produced in mammalian cells by both enzymatic and non-enzymatic pathways; the H_2_S formation by OSCs is due to their non-enzymatic reaction with GSH [16]. On this basis, the production of H_2_S from GaWs and GSGaWS extracts obtained using a low temperature was evaluated using the methylene blue assay (Figure 3). In particular, the H_2_S production, in the absence of reductant agents, from GSGaWS was higher than from GaWS extracts. This result is likely due to the presence of both GS conjugates and glutathione in the extract. The incubation in the presence of DTT increased, in a concentration- and time-dependent manner, the release of H_2_S by both of the WS extracts (Figure 4). These results may suggest H_2_S-production by compounds with sulfane sulfur, such as persulfides and polysulfides, or by S°-producing compounds, such as alkyl-cysteine disulfides [15].

**Figure 5:**
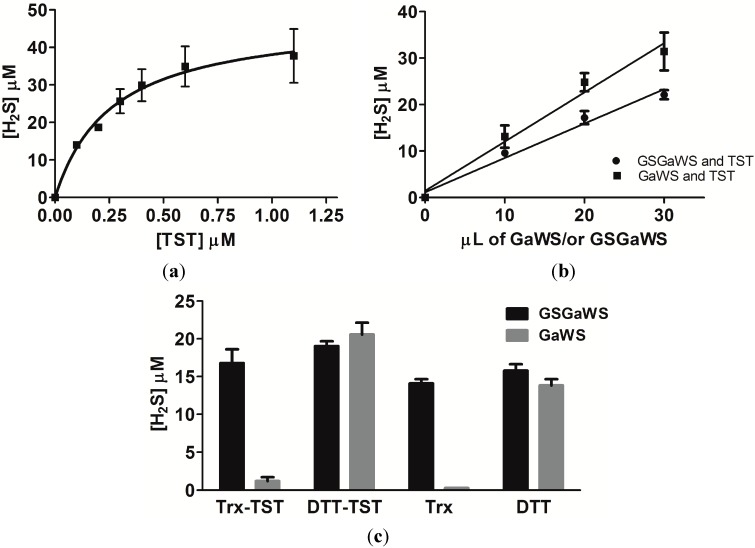
Catalysis of the H_2_S formation by TST enzyme. (**a**) H_2_S formation obtained using different concentrations of TST enzyme in the presence of 3 mM thiosulfate and 1 mM DTT; (**b**) using different concentrations of GaWS and GSGaWS in the presence of 0.22 μM TST enzyme; and (**c**) by 20 μL of GaWS (0.64 mg) and of GSGaWS (0.88 mg), in the presence or in the absence of 1.1 μM TST, with 4 mM DTT or with 4 μM Thioredoxin. Each bar represents ±SD of three experiments.

The human TST is one of the enzymes able to catalyze H_2_S formation, in the presence of thiosulfate and DTT [22]. Thus, TST could be involved in both the metabolism of OSCs and in the production of the gasotransmitter, H_2_S, in mitochondria. In order to study the ability of this enzyme to catalyze the release of H_2_S by garlic OSCs, the* in vitro* H_2_S formation by GaWS and GSGaWS extract was analyzed in the presence of recombinant TST from *A. vinelandii*. Firstly, the ability of this TST enzyme to catalyze the H_2_S formation, in the presence of 1 mM DTT and 3 mM thiosulfate, was demonstrated (Figure 5a). The hyperbolic curve might be due to an inhibition of the enzyme by the product. A small increase of the H_2_S-production by both of the WS extracts was observed in the presence of the TST enzyme and DTT and, in particular, in higher concentrations using GaWS extract (Figure 5b,c). These data may be in agreement with a possible role of TST in the metabolism of OSCs and in the formation of H_2_S at the mitochondrial level.

### 2.4. Thioredoxin as the Reductant in the Endogenous H_2_S Production from OSCs

The Trx⁄Trd system is an important endogenous antioxidant system in all living cells, in addition to the glutathione system, and often, high levels of Trx have been associated with cancer resistance to therapy [48,49]. In the present study, H_2_S formation by GSGaWS and GaWS extracts was tested using thioredoxin as the reducing agent (Figure 5c). GSGaWS only led to H_2_S releasing at a low concentration of thioredoxin (4 μM) in the presence or absence of TST. Thus, human TST, similarly to MST, can generate H_2_S reacting with thioredoxin [22,23], suggesting that the latter might be a physiologically-relevant partner of TST and MST for the production of the gasotransmitter. However, GSGa conjugates can spontaneously generate H_2_S by reaction with reduced thioredoxin, which can be the physiologic reductant of these conjugates in cells. Taken together, these results suggest that GSGaWS extracts could be a more stable and efficacious H_2_S-releasing solution than the simple garlic WS extracts for therapeutic applications in diseases associated with H_2_S deficiency. Moreover, these data are also in agreement with the previously-observed oxidation of the mitochondrial TST/Trx/Trd-system by a water-soluble garlic OSC [11]. Thus, this event could either reduce the antioxidant activity of the Trx on enzymes involved in relevant biological processes or increase the intracellular H_2_S formation.

### 2.5. Effects of the Garlic Water-Soluble Extracts on the Proliferation of the Human T-Cell Lymphoma Cell Line, HuT 78

**Figure 6:**
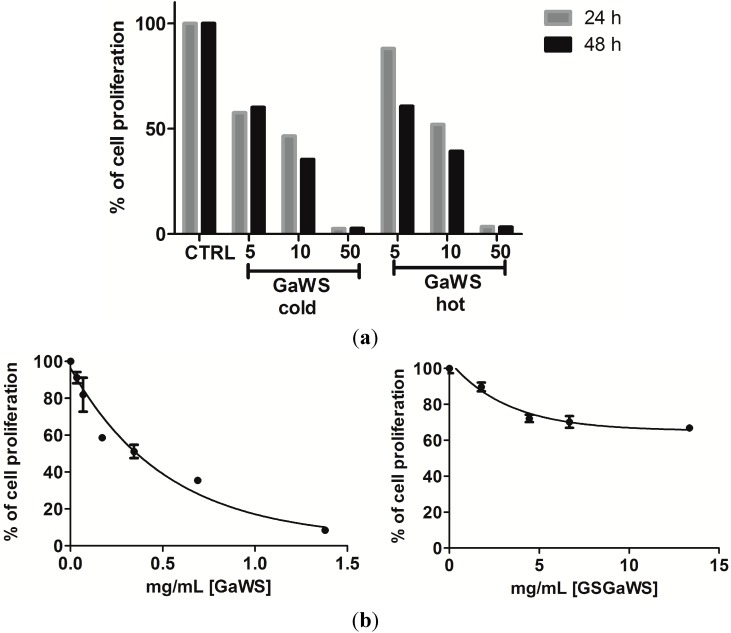

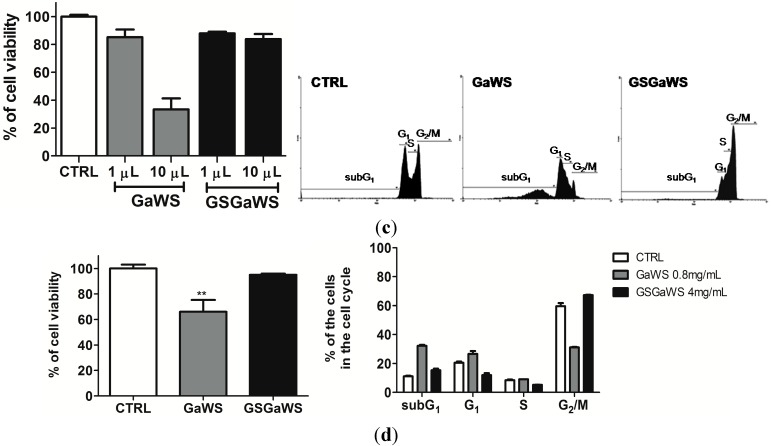
Dose-dependent inhibition of HuT 78 cell proliferation (**a**) after 24 h of treatment with cold and hot GaWS extracts and (**b**) after 24 h of treatment with cold GaWs and GSGa extracts. Trypan blue staining was used to differentiate viable cells. (**c**) The HuT 78 cell viability of untreated and treated cells with different concentrations (1 and 10 μL) of GaWS and GSGaWS extracts after 24 h of treatment and FACS analyses; (**d**) antiproliferative effects of the GaWS (0.8 mg/mL) and GSGaWS (4 mg/mL) stored seven months at −20 °C on HuT 78 cells and FACS analyses with the HuT 78 cell distribution at various stages of the cell cycle. *p*-values were 0.0112. ** p < 0.05, a significant antiproliferative effect with respect to the control using one-way ANOVA.

An important issue in cancer treatment is the therapeutic selectivity; not all cells are equally susceptible to the deleterious effects of the OSCs. In particular, neoplastic cells tend to be more susceptible to OSCs, suggesting that the uncontrolled proliferation of tumor cells may also be related to an incorrect functionality of the enzymes involved in sulfane sulfur metabolism and in the detoxification system. This possibility places the natural OSCs as potential ideal agents in anticancer therapy. Active sulfur metabolizing enzymatic systems could be beneficial to cells contributing the maintenance of low intracellular concentrations of reactive and toxic sulfur species, such as H_2_S, which, otherwise, in high levels, could induce apoptosis, as observed in neoplastic cells. The effects of the water-soluble garlic extracts on the growth of HuT 78 cells were investigated by the trypan blue dye exclusion assay, as well as by the MTT assay. About 30% and 70% of the HuT 78 cells were viable after 24 h of exposure, respectively, at 10 μL of GaWS (corresponding to 0.32 mg (d.w.)/mL) and GSGaWS (corresponding to 0.44 mg (d.w.)/mL) extracts (Figure 6a). A statistically-significant decrease in the number of viable cells at different concentrations of the extracts (from 1 to 10 μL) at 24 h, compared to the control, was observed (Figure 6c).

**Figure 7:**
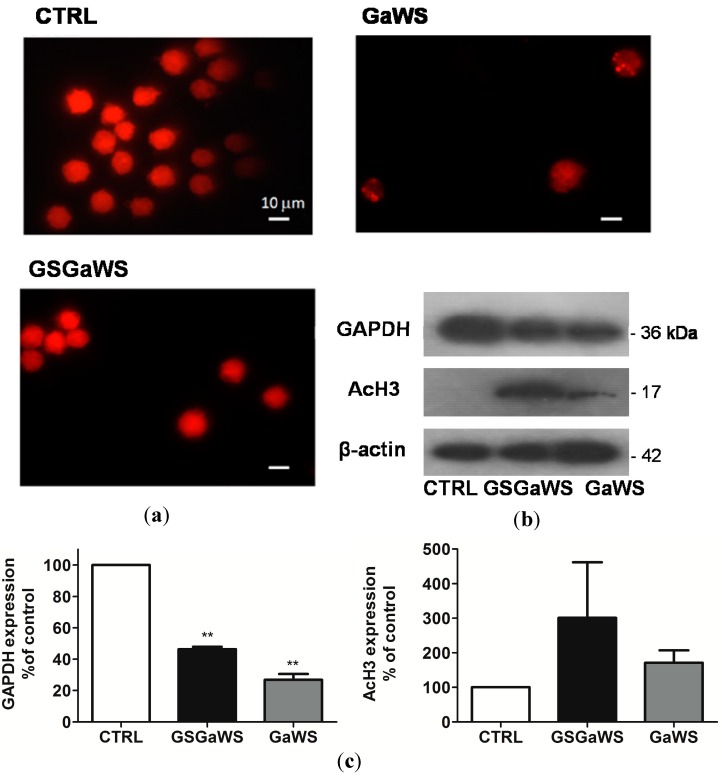
Fluorescence microscope images of the nucleus (**a**) stained with PI solution of the HuT 78 tumor cells after 24 h of incubation, untreated (CTRL) and treated with 0.32 mg (d.w.)/mL and 0.44 mg (d.w.)/mL of GaWS and GSGaWS extracts, respectively; (**b**) western blot analysis of the protein expression of the HuT 78 treated with 0.69 mg (d.w.)/mL and 6.67 mg (d.w.)/mL of GaWS and GSGaWS extracts, respectively. (**c**) Significant changes of the AcH3 and GAPDH expression were observed after both treatments. β-actin was used as the loading control. Western blot analysis was performed on a 15% SDS-PAGE with ~50 μg of total protein extracts. Results are indicative of three individual experiments. ** *p*-value = 0.0053 (<0.05).

Variations in the concentration-dependent manner of cell viability are shown in Figure 6b, and the apparent IC_50 _for the GaWS extract treatment was of 0.623 ± 0.023 mg/mL. Flow cytometric analysis of HuT 78 cells after 24 h of treatment with GaWS extract resulted in a significant increase in the fraction of subG_1_ over the control and a blockage in the G_1_/S phase (Figure 6c). By contrast, FACS analysis of HuT 78 cells after 24 h of treatment with GSGaWS extract resulted in a significant arrest of the cycle in the G_2_/M phase with a small increase of the subG_1_ fraction (Figure 6c). Similar effects on tumor cells were also observed using the garlic extracts stored for seven months at −20 °C (Figure 6d). This indicated that the cold garlic-WS extracts could preserve their biological activity in the long term. In Figure 7, fluorescence microscopy micrographs show the nucleus of non-treated and treated HuT78 tumor cells; it is possible to observe a decreased number of cells due to the treatments and the presence of pyknotic nuclei in the treated cells, especially after the treatment with GaWS. A higher and faster antiproliferative effect of the GaWs extract could be due to both the major cell permeability of the GaWs compounds and to their direct reaction with essential proteins, such as thioalkylation.

Antiproliferative effects of garlic compounds on cancer cells, such as cell cycle arrest in the G_2_/M phase, have been related to histone hyperacetylation [50,51]. Thus, histone H3 hyperacetylation in HuT78 cells was investigated after treatment with GaWS and GSGaWS extracts. A significant increase in the acetylated histone H3 form, especially after GSGaWS treatment, was observed (Figure 7b). Thus, the metabolic conversion of the GaWS and GSGaWS compounds, such as the presence of γ-glutamyl-S-allyl-L-cysteine, may play a pivotal role in generating intermediates with HDAC inhibitory activity and contribute to the overall garlic cancer chemoprotective properties [52,53].

Considering the ability of the garlic WS extracts to interact with the catalytic cysteine of the TST, we also investigated the effect on the expression of GAPDH, a key glycolytic enzyme characterized by the presence of a cysteine in the active site. The western blot analysis showed a down-regulation of the GAPDH expression after treatments that can be associated with the apoptosis induction and the cell cycle arrest. The housekeeping glycolytic enzyme, GAPDH, is also a key redox-sensitive protein, and it is largely affected by covalent modifications by oxidants at its highly reactive Cys152 residue. The protein is inhibited when it undergoes S-nitrosylation by nitric oxide (NO) [54,55], nitroalkylation by nitrated fatty acids [56], S-glutathionylation by glutathione [57], as well as from extensive oxidation by H_2_O_2_ or peroxynitrite [58,59]. These oxidative changes not only affect the glycolytic function, but also stimulate the participation of GAPDH in cell death processes [60]. Thus, we would like to propose that a thiolation/thioalkylation of this protein at the reactive Cys, due to allyl-sulfane sulfur compounds, might induce conformational changes of the protein, affecting its structural stability and degradation, similarly to what has been previously observed* in vitro* on the TST enzyme [11].

## 3. Experimental Section

### 3.1. OSCs Extraction Protocol

About 10 g of garlic cloves were cut in the presence or in the absence of 100 mM GSH, in 50 mM Tris-HCl buffer, pH 7.5. The cutting was performed at room temperature in about 10 min, in both extraction protocols, performed at both low and high temperatures. For the extraction at a low temperature, crushing was performed using liquid N_2_ (cold extraction), while in the case of hot extraction, the homogenates were put in a water bath at 100 °C for 15 min. After centrifugation of the homogenates, the fractions obtained (soluble and pellet) were separately treated. WS fractions were filtered with filters with a cut-off of 0.45 mm and then were ultra-filtrated at 4 °C. Ultra-filtered fractions were stored at −20 °C and analyzed for the molecular characterization by RP-HPLC, ^1^H-NMR, LC-MS and GC-MS, and the biological activity was assayed. RP-HPLC analyses were performed using mod. LC-10AVP (Shimadzu, Milan, Italy) with a Solvent B gradient (0–5 min, 0%; 5–55 min, 60%; 55–60 min, 60% and 65–85 min 90%), using 0.1% trifluoroacetic acid as Solvent A, 80% CH_3_CN and 0.1% trifluoroacetic acid as Solvent B and a C18 column (CPS Analitica, 150 × 4.6 mm, 5 μm). The elute was monitored at 220 nm by a UV detector (Shimadzu, Milan, Italy), and the samples were then analyzed by C4T (Colosseum Combinatorial Chemistry Centre for Technology, University of Rome Tor Vergata, Italy) using LC/ESI-MS spectroscopy with a C18 column. High-resolution ^1^H-NMR experiments were performed at 25 °C using 400 MHz (Bruker AVANCE spectrometer, Italy) and using a Bruker Win-NMR software package for the deconvolution of the signals.

### 3.2. H_2_S Assay of the Garlic Extracts

This method consists of the reaction between sulfide and N,N-dimethyl-p-phenylenediamine sulfate in the presence of the oxidizing agent, Fe^3+^, in hydrochloric acid to form methylene blue, involving a 1:2 stoichiometry [61]. Briefly, reaction mixtures were incubated at 37 °C for 30 min, and the reaction was terminated by the addition of 20 μL of Solution I (20 mM *N*',*N*'-dimethyl-p-phenylenediamine dihydrochloride in 7.2 M HCl) and 20 μL of Solution II (30 mM FeCl_3_ in 1.2 M HCl). After incubation for 10 min and mixing at room temperature, samples were analyzed spectrophotometrically at 670 nm. Experiments in the presence of TST enzyme were performed using 0.12–1.1 µM TST enzyme; then, the standard curve for H_2_S was prepared using 0–350 μM Na_2_S as the source of H_2_S. After adding 1 mM DTT of 50 mM Tris-HCl buffer, pH 7.4, the mixtures were incubated at 37 °C for 30 min while shaking on a rotary shaker to facilitate the release of H_2_S gas from the aqueous phase. The following steps were the same as described above. The results were plotted using GraphPad Prism version 5.0 for Windows (GraphPad Software, San Diego, CA, USA).

### 3.3. TST Assay

TST activity was measured by the discontinuous colorimetric assay described by Sörbo [39], where the production of thiocyanate from thiosulfate and cyanide was followed at 460 nm using a Perkin-Elmer spectrometer.

### 3.4. Cell Proliferation and Vitality Assay

The human T-cell lymphoma cell line, HuT 78, was purchased from the ISS (Istituto Superiore di Sanità, Italy). HuT 78 (0.2 × 10^6^) cells were pre-incubated for 24 h in RPMI medium 1640 (GIBCO, Monza, Italy) in the presence of 1% glutamine, 10% heat-inactivated FSC and antibiotics (1% penicillin and streptomycin sulfate) at 37 °C in air supplemented with 5% CO_2_. HuT 78 cells were treated with different concentrations of garlic extracts and then monitored for 24 and 48 h. The cells were then collected and counted after trypan blue staining (0.4% trypan blue solution, Sigma-Aldrich, Milan, Italy) by optical microscopy using a Thoma chamber. The rates of growth inhibition with respect to the control culture taken as 100% growth were calculated, and the percent of cell viability was also obtained through performing the MTT assay [62]. The HuT 78 cells were fixed with 4% paraformaldehyde for 20 min followed by incubation for 15 min with propidium iodide (Sigma-Aldrich, Milan, Italy) solution and washing with PBS buffer. The cells were mounted on slides and analyzed by fluorescence microscopy using a microscope (Nikon, Filter) and Lucia G version 4.61 software.

### 3.5. Cell Cycle Analysis

The cell cycle distribution of HuT 78 cells was measured by flow cytometry, and harvested cells (about 0.5 × 10^6^ cells) were stained with 50 µg/mL propidium iodide (Sigma-Aldrich, Milan, Italy) in PBS buffer using 0.1% Triton X-100 and 1 mg/mL sodium citrate. Subsequently, they were immediately analyzed using a flow cytometer FACSCalibur (Beckton and Dickinson, San Jose, CA, USA), and the percentage of cells in each phase of the cell cycle was evaluated according to Nicoletti* et al.* [63].

### 3.6. Western Blot Analysis

Western blot analyses were performed as described previously [11]. Protein concentrations were measured with the bicinchoninic protein assay (Sigma-Aldrich, Milan, Italy), and equal amounts of protein were loaded into lanes of 15% polyacrylamide-SDS gels. The gels were electrophoresed, followed by the transfer of the protein to a PVDF membrane (Sigma-Aldrich, Milan, Italy). The membrane was then blocked and probed with primary antibodies (Ab-β-actin mouse, Ab-GAPDH rabbit, Ab-AcH3 rabbit) (Sigma-Aldrich, Milan, Italy) overnight at 4 °C. Immunoblots were next processed with secondary antibodies (Sigma-Aldrich, Milan, Italy) for 2 h at room temperature. Immunoblots were finally probed with a Super Signal West Pico kit (Thermo Scientific, Rodano- Milan, Italy) to visualize the signal, followed by exposure to X-ray film (Kodak, Sigma-Aldrich, Milan, Italy).

### 3.7. Statistical Analysis

Cell survival, IC_50_ and tumor volumes are expressed as the mean ± standard error (SEM). For *in vitro* studies, cell survival of both non-treatment and treatment groups was analyzed using the one-way ANOVA test or the Student’s *t*-test. *p*-values <0.05 were considered significant.

## 4. Conclusions

Although the purification of active compounds from plant extracts seems to be the logical way to obtain higher biological activity, several problems may raise during the purification steps: the higher toxicity of the active compounds at lower doses [64]; the high costs; and the technology associated with the purification steps. These can be financial restraints in certain communities and decrease the affordability of the treatment. Moreover, some plant extracts may contain several active compounds with better bioavailability [65] and with synergistic effects with other compounds present in the same extract. Thus, in the present work, a new protocol of extraction of garlic-OSC at low temperatures and the production of OSC-glutathionyl conjugates during the extraction were optimized. Water-soluble compounds are more stable and less toxic than oil-soluble compounds and offer better intestinal absorption [66,67]. Accordingly, the characterization and the analysis of some biological properties of WS extracts were herein performed. While the GaWS extract leads to a thioalkylation of the catalytic cysteines of the enzymes, such as observed in the case of the TST enzyme, and induce apoptosis in the cancer HuT 78 cell line, the GSGaWS extract does not induce a direct inhibition of the enzyme with catalytic cysteine and leads to a cell cycle arrest in the G_2_/M phase. Both extracts are able to release H_2_S* in vitro*, but the results suggest that the formation of GSGa conjugates may lead to an increased production of H_2_S* in vivo* in the presence of reduced Trx. Thus, GSGaWS extract may be promising for the formulation of H_2_S-donating pro-drugs. These pro-drugs are under development for the potential treatment or prevention of conditions like arthritis, cardiovascular, urological, neuro-inflammatory and ophthalmological diseases [16,68,69]. Only limited studies have reported the effect of H_2_S donors on cancer cells* in vitro* and on tumor progression* in vivo* [70,71,72,73,74], and some of the mechanisms of action of these novel agents have been addressed [75,76]. Some slow-releasing H_2_S donors inhibit tumor growth both* in vitro* and* in vivo* by a combination of cell cycle arrest and apoptosis promotion, while no cell death was apparent in non-cancer cells [75]. Thus, although many details of the underlying molecular mechanism of action of the garlic extracts, investigated here, remain to be elucidated, the observed effects on the control of gene expression by histone-hyperacetylation and the down-expression of the glycolytic enzyme, GAPDH, in cancer cells seems relevant for further exploring the use of these extracts as adjuvants in anticancer therapy.

## Supplementary Materials

Supplementary materials can be accessed at: http://www.mdpi.com/1420-3049/20/01/1731/s1.

## Abbreviations

2-PTS: sodium-2-propenyl thiosulfate
CBS: cystathionine-β-synthase
CSE: cystathionine-γ-lyase
E: de-persulfurated form of TST
ES: persulfurated form of TST
FACS: fluorescence-activated cell sorting
GSH: glutathione
GAPDH: glyceraldehyde-3-phosphate dehydrogenase
GaWS: water-soluble garlic extract
GSSA: S-allyl-mercapto-glutathione
GSGaWS: water-soluble glutathione-garlic extract
GS-OSCs: glutathione-garlic organosulfur conjugates
GST: glutathione-S-transferase
RP-HPLC: reversed-phase high-performance liquid chromatography
HuT 78: the human T-cell lymphoma cell line HuT 78
MST: 3-mercaptopyruvate sulfurtransferase
MTT: 3-(4,5-dimethylthiazol-2-yl)-2,5-diphenyltetrazolium bromide
OSCs: organosulfur compounds
PI: propidium iodide
SAC: S-allylcysteine
Trx: thioredoxin
Trd: thioredoxin reductase
TST: cyanide:thiosulfate sulfurtransferase
