# Supplementary material for: Glutathione-Garlic Sulfur Conjugates: Slow Hydrogen Sulfide Releasing Agents for Therapeutic Applications
Source: Molecules. 2015 Jan 20;20(1):1731–50. doi: 10.3390/molecules20011731 (PMC6272329; doi:10.3390/molecules20011731)
Supplement: Supplementary file 1 [file molecules-20-01731-s001.pdf]

Supplementary Materials

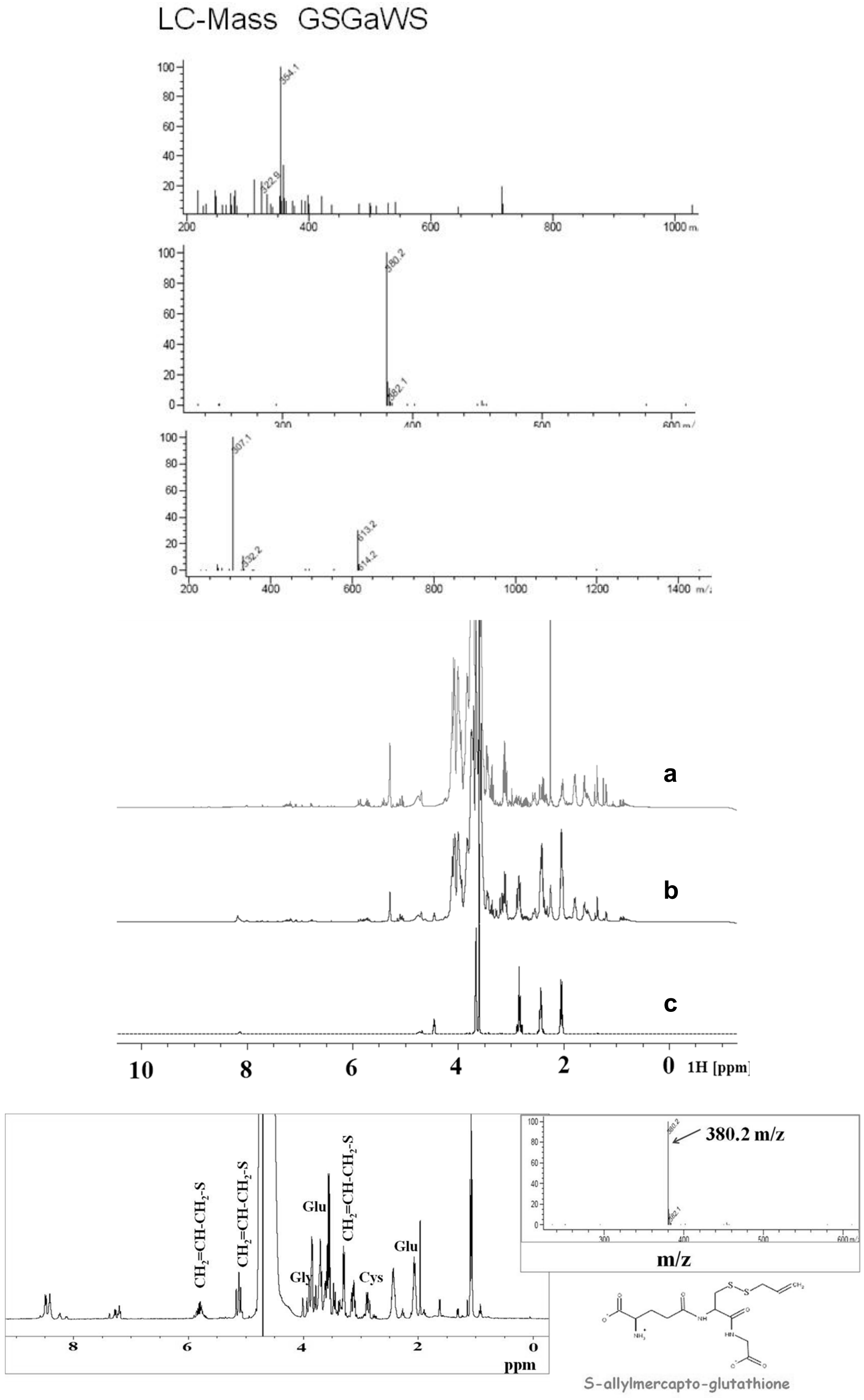

Figure S1. 1D ^{1}H-NMR spectra in 30% D2O and LS-MS analysis of the peaks obtained by RP-HPLC of the cold GaWS or GSGaWS fractions.

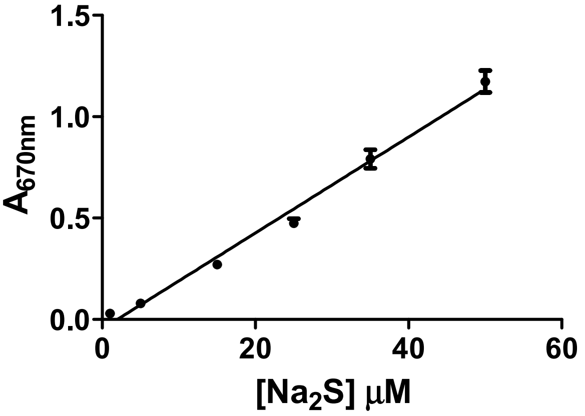

Figure S2. Calibration curve of H_2S formation by the methylene blue assay, obtained at different concentrations of Na_2S. Absorbance: 670 nm. Each bar represents ±SD of three experiments.

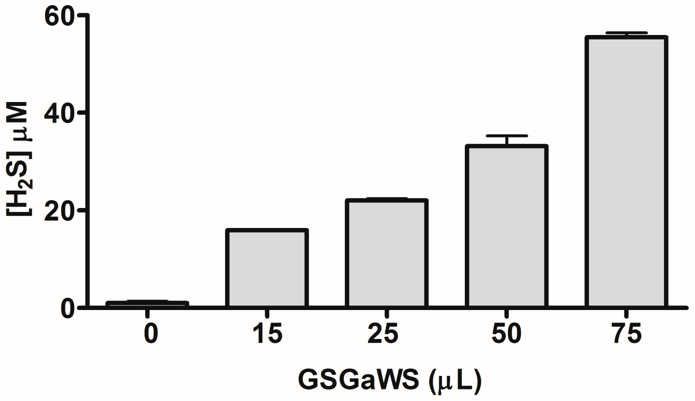

Figure S3. H_2S-release at different concentrations of GSGaWS extract in the presence of 0.12 μM TST.
